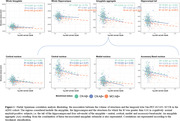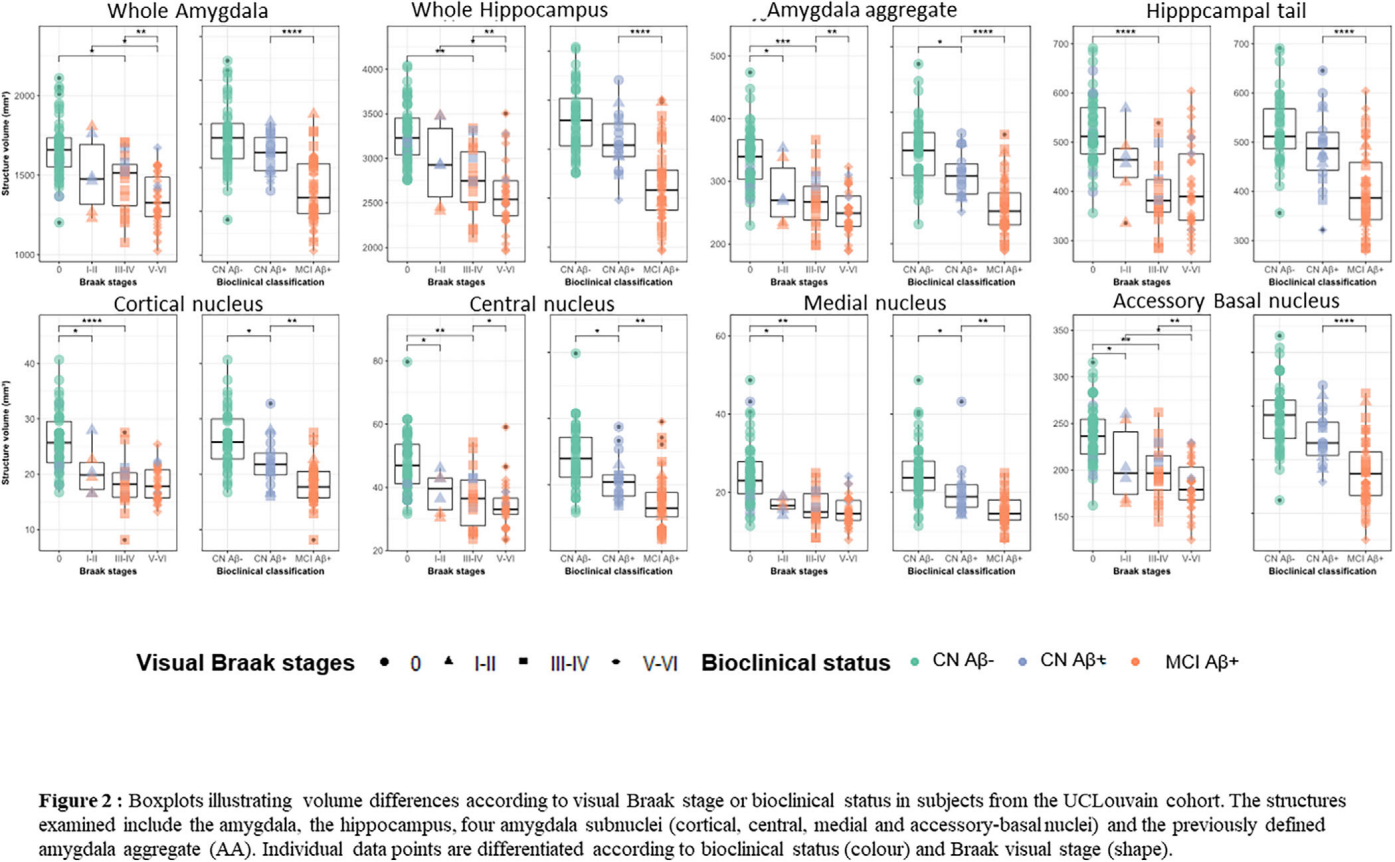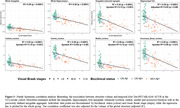# The atrophy of specific amygdala subnuclei is associated with temporal tauopathy in preclinical AD individuals

**DOI:** 10.1002/alz.093684

**Published:** 2025-01-09

**Authors:** Yasmine Salman, Thomas Gérard, Lara Huyghe, Lise Colmant, Lisa Quenon, Vincent Malotaux, Emilien Boyer, Adrian Ivanoiu, Renaud Lhommel, Laurence Dricot, Bernard J Hanseeuw

**Affiliations:** ^1^ Institute of Neuroscience, UCLouvain, Brussels Belgium; ^2^ UCLouvain ‐ Institute of Neuroscience, Brussels Belgium; ^3^ Institute of Neuroscience ‐ UCLouvain, Brussels Belgium; ^4^ Department of Neurology, Saint‐Luc University Hospital, Brussels Belgium; ^5^ Nuclear Medicine Department, Saint‐Luc University Hospital, Brussels Belgium

## Abstract

**Background:**

In AD, tauopathy and atrophy start in the mesiotemporal lobe, including the amygdala‐hippocampal complex. Until recently, subnuclei and subfields within these structures were indistinguishable in‐vivo. FreeSurfer 7.0 now enables their segmentation. Our goal was to study hippocampal and amygdala subregions’ atrophy in preclinical AD to assess whether specific subregion atrophy could indicate early tauopathy at the preclinical stage.

**Methods:**

We conducted an exploratory study in the ADNI3 cohort including 144 amyloid‐positive cognitively‐normal adults (Aß+CN) with 3DT1‐MRI and [18F]AV‐1451 Tau‐PET data. We identified hippocampal and amygdala subregions explaining at least 1% of the variance of the Tau‐PET signal from the temporal meta‐ROI defined by Jack (2017). We validated these results with our in‐house data, including 112 individuals with 3DT1‐MRI and [18F]MK6240 Tau‐PET scans. Groups were categorized by visual Braak‐stage or bioclinical classification based on cognition (CN/MCI) and amyloid status (Aß‐/Aß+).

**Results:**

In ADNI3, amygdala and hippocampal volumes were not associated with temporal tauopathy in Aß+CN (R²<0.001). However, four amygdala subnuclei (cortical, central, medial, and accessory‐basal nuclei) out of nine and the hippocampal tail (out of twelve subfields) each explained over 1% of the variance in temporal tauopathy in Aß+CN (one‐tail p<0.10). These tau‐associated subnuclei were pooled into an amygdala aggregate whose volume was significantly associated with temporal tauopathy in Aß+CN, even after adjusting for amygdala volume. In Aß+MCI (n=132), global structure volumes were significantly associated with temporal tauopathy, with subnuclei atrophy adding no explanatory power (Figure 1). Within our in‐house data, individuals with early Tau‐PET signal (Braak I‐II) had similar global amygdala and hippocampal volumes than Braak 0 individuals. However, the tau‐associated amygdala aggregate generated in ADNI3 was significantly smaller in Braak I‐II individuals. Similarly, only the aggregate was significantly smaller in Aß+CN compared to Aß‐CN (Figure 2). These results remained significant after adjusting for global structures (Figure 3).

**Conclusion:**

We identified four amygdalae subnuclei whose atrophy is earlier than global amygdala. Atrophy in these subnuclei is associated with temporal tauopathy in preclinical AD, distinguishing visual Braak‐stage I‐II from Braak‐stage 0 individuals. Measuring amygdala subnuclei volumes in older adults is a promising approach to identify individuals at‐risk of progression to clinical AD.